# Comparative analysis of cutaneous bacterial communities of farmed* Rana dybowskii* after gentamycin bath

**DOI:** 10.7717/peerj.8430

**Published:** 2020-01-20

**Authors:** Jia Bie, Qing Tong, Xiaoning Liu, Xianhao Zhang, Hongbin Wang

**Affiliations:** Northeast Agricultural University, Harbin, China

**Keywords:** *Rana dybowskii*, Gentamicin, High-throughput sequencing, Cutaneous bacterial community

## Abstract

**Introduction:**

Pathogenic bacteria limit the success of *Rana dybowskii* breeding. Gentamicin is used to treat *R. dybowskii* disease. To understand the effects of gentamicin on the composition and structure of the cutaneous bacterial community of *R. dybowskii*, three groups (control, gentamicin and recovery) were established in this study.

**Materials & Methods:**

The V3–V4 hypervariable region of the 16S rRNA gene was analyzed in samples by high-throughput sequencing. Alpha diversity and beta diversity were evaluated to compare the cutaneous bacterial community diversity.

**Results:**

A total of 1,159,668 valid sequences and 3,132 operational taxonomic units (OTUs) were obtained from these three experimental groups. The number of OTUs obtained in the control group, gentamicin group and recovery group were 2,194, 2,288, and 2,047, respectively, and the number of shared OTUs was 1,313. The alpha diversity of the cutaneous bacterial community was not significantly affected by gentamicin, while beta diversity was significantly affected.

**Discussion & Conclusions:**

The effect of a gentamicin bath on relative species abundance was greater than the effect on the species composition. The changes in Proteobacteria, *Acinetobacter*, and *Chryseobacterium* were significant, and reductions were observed after the recovery period. Six potentially pathogenic genera were detected, including *Aeromonas, Citrobacter, Chryseobacterium, Pseudomonas, Staphylococcus,* and *Streptococcus*. Among them, *Aeromonas* and *Chryseobacterium* were significantly inhibited by the gentamicin bath. The results of this study provide a theoretical basis for the application of gentamicin in *R. dybowskii* breeding.

## Introduction

*Rana dybowskii* is an important amphibian species in northeast China and has been assessed as a near-threatened species by the Red List of China’s Vertebrates (Jiang et al., 2016). *Rana dybowskii* is a rare frog species with medicinal value, nutritional value and a variety of health effects (Liang et al., 2008; Wu et al., 2008; Wang et al., 2013). Oviductus ranae (OR) is a traditional and highly safe animal-based Chinese medicine that has been listed in the Chinese Pharmacopoeia since 1985 (Zhang et al., 2017). To protect and rationally use *R. dybowskii*, extensive studies of its ecology, artificial domestication and utilization have been performed (Tong et al., 2018). Since development of the *R. dybowskii* breeding industry more than 30 years ago, research has focused on improving the artificial breeding process. At present, artificial breeding technologies for *R. dybowskii* are still immature. A main issue for successful application is insufficient methodology for preventing and treating diseases. *Rana dybowskii* is susceptible to red-leg syndrome, gastroenteritis and rotten-skin disease (Pasteris, Bühler & Nadermacías, 2006; Shu et al., 1997). The pathogenic bacteria causing these diseases are mostly opportunistic, with an onset season from June to September, which is closely related to the health of the host and the cleanliness of the environment (Xu et al., 2017). Generally, only insects, such as yellow mealworm (*Tenebrio molitor*), can be used as feed in artificial breeding because *R. dybowskii* only prey on active food. Food additives, antibiotics, etc. cannot be added to the food, making it difficult to prevent and control diseases. Therefore, disinfectants and antibiotics are sprayed in the culture environment for disease prevention and treatment. In addition, for individuals, medicated baths are used to control the causal agents of the diseases (Chinnadurai & Kane, 2014). At present, disease is a main factor limiting development of the *R. dybowskii* aquaculture industry (Bai, 2009). Thus, further research on the efficacy of using a gentamicin bath may help with this problem.

Antibiotics are widely used in aquaculture and can be added directly to cultured pond water. Studies have shown that gentamicin is effective for serious gram-negative bacterial infections (Wiedenhoft, Hayashi & Coffin, 2017). Pathogenic bacteria, such as *Arcobacter butzleri* (Rathlavath et al., 2017), *Staphylococcus aureus* (Awad et al., 2017), *Pseudomonas aeruginosa* (Lamari, Chakroun & Rtimi, 2017), *Acinetobacter* (Jie et al., 2017) and *Rahnella aquatilis* (Lü et al., 2017), which cause diseases in aquatic animals, are all sensitive to gentamicin. *Flavobacterium psychrophilum* is a serious pathogen of salmonids. Biosecurity measures and antimicrobial agents remain the only available methods to control diseases caused by *F. psychrophilum* owing to the lack of effective vaccine preparations (Van et al., 2017). Gentamicin has a broad antibacterial spectrum, which also affects beneficial microorganisms. Studies of the percutaneous absorption of gentamicin have shown that a dose of 1 mg/L results in a low serum drug concentration (Riviere, Shapiro & Coppoc, 2010), whereas a bacteriostatic effect is obtained at a dose of 16 mg/L *in vitro* (Sullam et al., 1985). In this experiment, gentamicin was used at a dose of 20 mg/L, which is close to the dose used for fish seedlings in aquaculture.

Skin bacterial community plays an important role in the host immune response and its diversity can affect the activity of pathogenic bacteria (Sabino-Pinto et al., 2016; Becker et al., 2014). The microbiome of amphibian skin can change with the environment, which may further lead to an increased relative content of opportunistic pathogens, thus endangering the health of the host (Lokesh & Kiron, 2016). To better understand the effects of a gentamicin bath on the cutaneous bacterial community of *R. dybowskii*, some *R. dybowskii* were collected from a farm in Northeast China. The samples were subjected to a gentamicin bath treatment and the cutaneous bacterial communities were examined and compared with those of the control and recovery groups. The recovery group was used to observe the change in the cutaneous bacterial community after the gentamicin bath. However, the results of this study may be different from those for the medicated bath in the farm because the bath treatment was performed in the laboratory. In this paper, the 16S rRNA gene was sequenced by high-throughput sequencing to determine the composition and structure of the cutaneous bacterial community in samples after treatment by a gentamicin bath, after the recovery period and in the control group. The species composition and relative abundance of cutaneous bacterial communities, alpha diversity and differences in beta diversity were analyzed.

## Materials and Methods

### Sample collection

*Rana dybowskii* (*n* = 21) were collected from a farm in Huanan County (46°44′54″N, 130°69′32″E; 80 m alt) in August 2017 (weight, 18.07 ± 0.39 g; male-to-female ratio of 3:4). The experimental individuals were divided into three groups: gentamicin group (*n* = 7), recovery group (*n* = 7) and control group (*n* = 7). The seven individuals in the gentamicin group were treated with a gentamicin (E003632; Sigma, St. Louis, MO, USA) solution bath (20 mg/L) for 7 days. The seven individuals in the recovery group were treated with a gentamicin solution bath for 7 days (the same as the gentamicin group) and then they were fed for an additional 7 days after the end of the gentamicin solution bath. The control group was given a distilled water bath for only 7 days.

A 1.8-cm bath depth was selected to allow the liquid to immerse 1/3 of the body of the frog. To achieve better results with respect to the percutaneous absorption of drugs, the depth of the medicated bath should be determined according to the size of the individual *R. dybowskii* (Riviere, Shapiro & Coppoc, 2010). The solution used in the medicated bath was 2 L (1/3 of the volume of the frog), and the duration of the medicated bath was 1 h, once a day. The individuals were housed in plastic boxes (43.0 × 32.0 × 27.7 cm) in the laboratory. The bottom of each box was covered with an aqueous pad to ensure that the skin of the frog was moist. The individuals in the different groups were housed in separate boxes. The breeding temperature was 16 °C. Frogs were fed *Tenebrio* daily and freely (added as necessary to ensure that there was always food available). All frogs were housed in the same way, except during the one-hour medicated bath.

For this study, there were seven individuals in each group. All individuals were sacrificed to facilitate collection of both skin and gut samples (the gut samples were used for another study). Next, the skin of the back, venter surface and limbs was peeled off and collected. The skin samples were loaded into 5-mL sterile tubes and cryopreserved at −80 °C.

### DNA extraction and PCR amplification

Genomic DNA extraction procedure was performed using the FastDNA® Spin Kit for Soil (MP Biomedicals, Santa Ana, CA, USA) as per the manufacturer’s instructions. For each sample, the V3–V4 hypervariable region of the bacterial 16S rRNA gene was amplified with primers 338F (5′-ACTCCTACGGGAGGCAGCAG-3′) and 806R (5′-GGACTACHVGGGTWTCTAAT-3′) using a thermocycler PCR system (GeneAmp 9700: ABI, Foster, City, CA, USA). The PCRs were conducted using the following program: 3 min of denaturation at 95 °C; 27 cycles of 30 s at 95 °C, 30 s for annealing at 55 °C and 45 s for elongation at 72 °C; and a final extension at 72 °C for 10 min. PCRs were performed in triplicate using a 20 µL mixture containing 4 µL of 5 × FastPfu Buffer, 2 µL of 2.5 mM dNTPs, 0.8 µL of each primer (5 µM), 0.4 µL of FastPfu Polymerase, 0.2 µL of BSA and 10 ng of template DNA. The resulting PCR products were extracted from a 2% agarose gel, further purified using the AxyPrep DNA Gel Extraction Kit (Axygen Biosciences, Union City, CA, USA) and quantified using QuantiFluor™-ST (Promega, Madison, WI, USA) according to the manufacturer’s protocol.

### Illumina MiSeq sequencing

Purified amplicons were pooled in equimolar concentrations and paired-end sequenced (2 × 300) using an Illumina MiSeq platform (Illumina, San Diego, CA, USA) according to standard protocols.

Raw fastq files were demultiplexed, quality-filtered using Trimmomatic and merged using FLASH with the following criteria. (i) Reads were truncated at any site with an average quality score <20 over a 50-base pair (bp) sliding window. (ii) Primers were exact matches, allowing 2 nucleotide mismatches, and reads containing ambiguous bases were removed. (iii) Sequences whose overlap was longer than 10 bp were merged according to their overlap.

Operational taxonomic units (OTUs) were clustered with a 97% similarity cutoff using UPARSE (http://drive5.com/uparse/) and chimeric sequences were identified and removed using UCHIME. The taxonomic assignments for each 16S rRNA gene sequence were obtained by the RDP Classifier algorithm (version 2.2: http://sourceforge.net/projects/rdp-classifier/) against the SILVA (Release119: http://www.arb-silva.de) 16S rRNA gene database using a confidence threshold of 70% (Li et al., 2016). A total of 1,159,668 valid sequences and 511,762,953 bp were obtained by sequence filtration and double-stitched splicing, and the average sequence length was 441 bp. After subsampling each sample to an equal sequencing depth (23,863 reads per sample) and clustering, 3,132 OTUs at 97% identity were obtained.

### Statistical analysis

MOTHUR (version v.1.30.1: http://www.mothur.org/wiki/Schloss_SOP#Alpha_diversity) was used to create rarefaction curves and alpha diversity indices. ANOVA, followed by Tukey’s test, was used to analyze the difference in alpha diversity indices. Bray Curtis distance matrices were used for the ANOSIM analysis and to calculate beta diversity. Results were visualized by a principal coordinates analysis (Rebollar et al., 2016). Wilcoxon rank-sum tests were used to analyze the relative abundance of phyla and genera in the two groups. The Kruskal–Wallis H test was used to test for significant differences between groups. The FDR method was used to perform multiple test corrections on p-values (Benjamini & Hochberg, 1995) and Scheffer was used to calculate the confidence interval (CI).

### Potentially pathogenic genera

To understand the potentially pathogenic bacteria on the skin of *R. dybowskii*, several bacteria were selected for comparison. Potentially pathogenic genera associated with red-leg syndrome in amphibians include *Aeromonas*, *Citrobacter*, *Chryseobacterium*, *Edwardsiella*, *Proteus*, *Pseudomonas*, *Staphylococcus* and *Streptococcus* (Weng, Yang & Wang, 2016). The Student’s *t*-test was used to evaluate differences in potentially pathogenic genera.

### Ethical approval

Before sample collection, all animal protocols were approved by the Institutional Animal Care and Use Committee (IACUC) at the Northeast Agricultural University, China. Northeast Agricultural University was approved to take samples at the farm by the owner of the farm, Fenglin Gao. All experiments were performed in accordance with approved guidelines and regulations. IACUC#2015-035.

## Results

In this study, the V3–V4 hypervariable region of the bacterial 16S rRNA gene was sequenced to determine the composition and abundance of the bacterial community in *R. dybowskii* using high-throughput sequencing.

### Sample sequencing data information

In total, 21 samples were included in the three study groups. After clustering at a threshold of 97% similarity, the total number of OTUs obtained was 3,132, belonging to 1,766 species, 980 genera, 429 families, 219 orders, 107 classes and 49 phyla. The rarefaction curve tended to be flat, indicating that the samples were sequenced in a reasonable quantity and that the obtained community reflected the vast majority of the actual species (Fig. S1).

### Alpha diversity

In community ecology, alpha diversity can reflect the abundance and diversity of microbial communities, including a series of statistical indexes of the abundance and diversity of species in the community. Sobs, which represents the actual number of observed OTUs in each sample, confirmed that the species richness was higher in the gentamicin group than in the other groups (Table 1). The coverage index reflects whether the sequencing results accurately reflect the microorganisms in the sample. The coverage index for every sample was greater than 99% (Table 1), indicating that the sequencing results were credible. The Chao and ACE indices are used to estimate the total number of species and reflect the abundance of the bacterial community. There were no significant differences in the Chao and the ACE indices among the control, gentamicin and recovery groups (Chao, *p* = 0.852; ACE, *p* = 0.764). The Shannon and Simpson indices reflect the diversity of the bacterial community. There were no significant differences in the Shannon and the Simpson indices among the three groups (Shannon, *p* = 0.759; Simpson, *p* = 0.088).

**Table 1:** Sequence information and alpha diversity metrics for different samples (M ± SD).

Groups	Sequence Number	Sequence length	Sobs	coverage %
control	47430 ± 5552	441 ± 2	901 ± 156	99.47 ± 0.13
gentamicin	51076 ± 10943	439 ± 1	986 ± 172	99.47 ± 0.18
recovery	67161 ± 11429	441 ± 2	877 ± 345	99.31 ± 0.25

### Beta diversity analysis and shared OTUs

**Figure 1:**
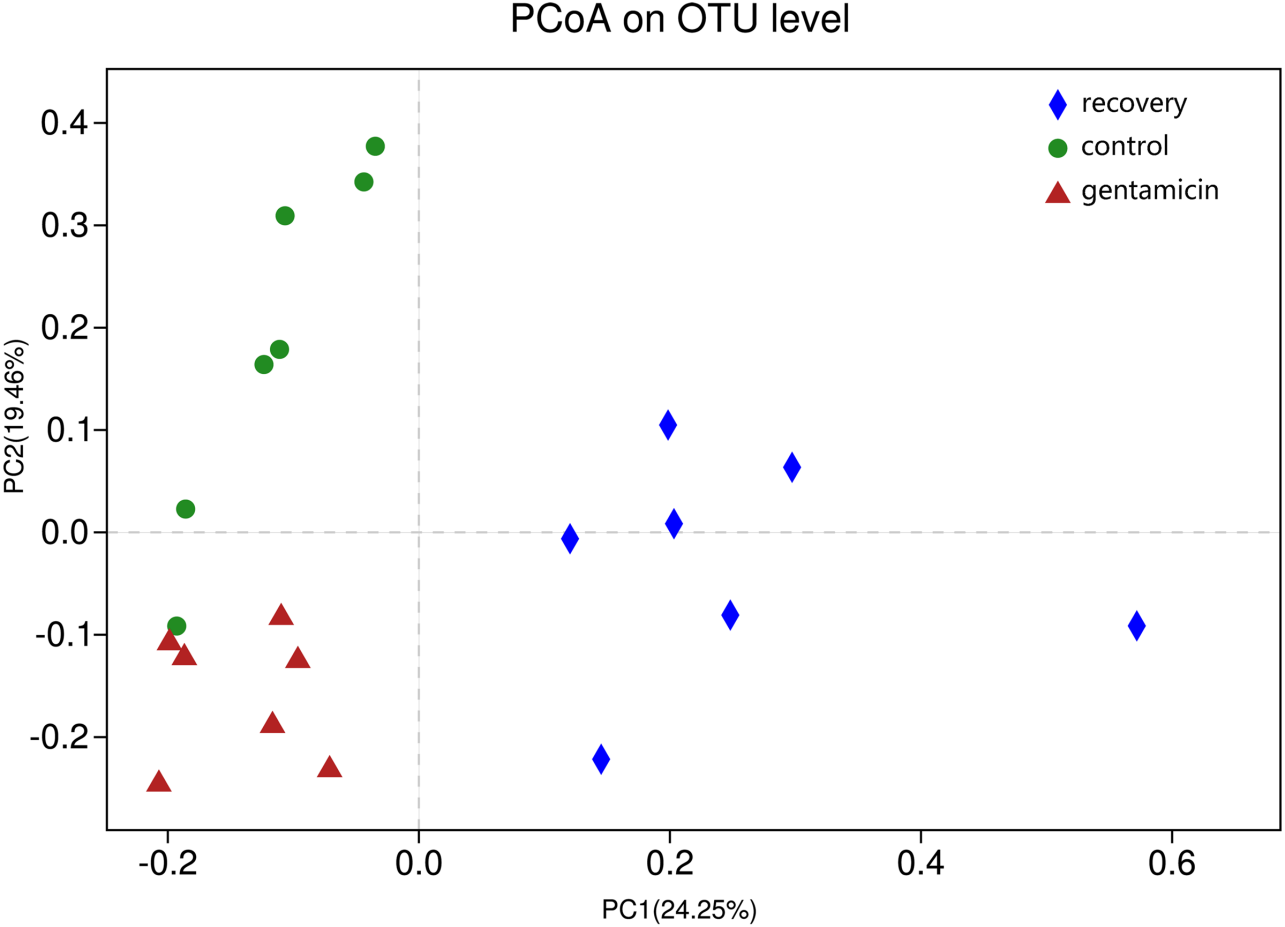
PCoA at the OTU level. PCoA plot depicting the clustering of bacterial communities in the three groups, showing differences in the sample community composition.

ANOSIM indicated that the differences between groups were greater than the differences within groups, and the difference was significant (*R* = 0.623, *p* = 0.001). The analysis showed that the grouping is meaningful. Beta diversity analyses using the Bray–Curtis distance algorithm indicated differences between the bacterial communities of the control, gentamicin and recovery groups. Clustering was evaluated by principal coordinate analysis (PCoA); the control, gentamicin and recovery groups exhibited separation along the principal coordinates (Fig. 1).

There were 2,194 OTUs in the control group, 2,288 in the gentamicin group, 2,047 in the recovery group and 1,313 shared OTUs. Although these OTUs were present in most samples, their abundances differed between groups. The shared OTUs accounted for 59.85% of OTUs in the control group, 57.39% in the gentamicin group and 64.14% in the recovery group. There were 13 shared OTUs with a high abundance (accounting for more than 1% of the total OTUs). These 13 shared OTUs belonged to Firmicutes (OTU3049), Bacteroidetes (OTU2686, OTU75, OTU843, and OTU1405), Proteobacteria (OTU2510, OTU830, OTU2410, OTU115, OTU2484, and OTU815) and Actinobacteria (OTU1735 and OTU963).

**Figure 2:**
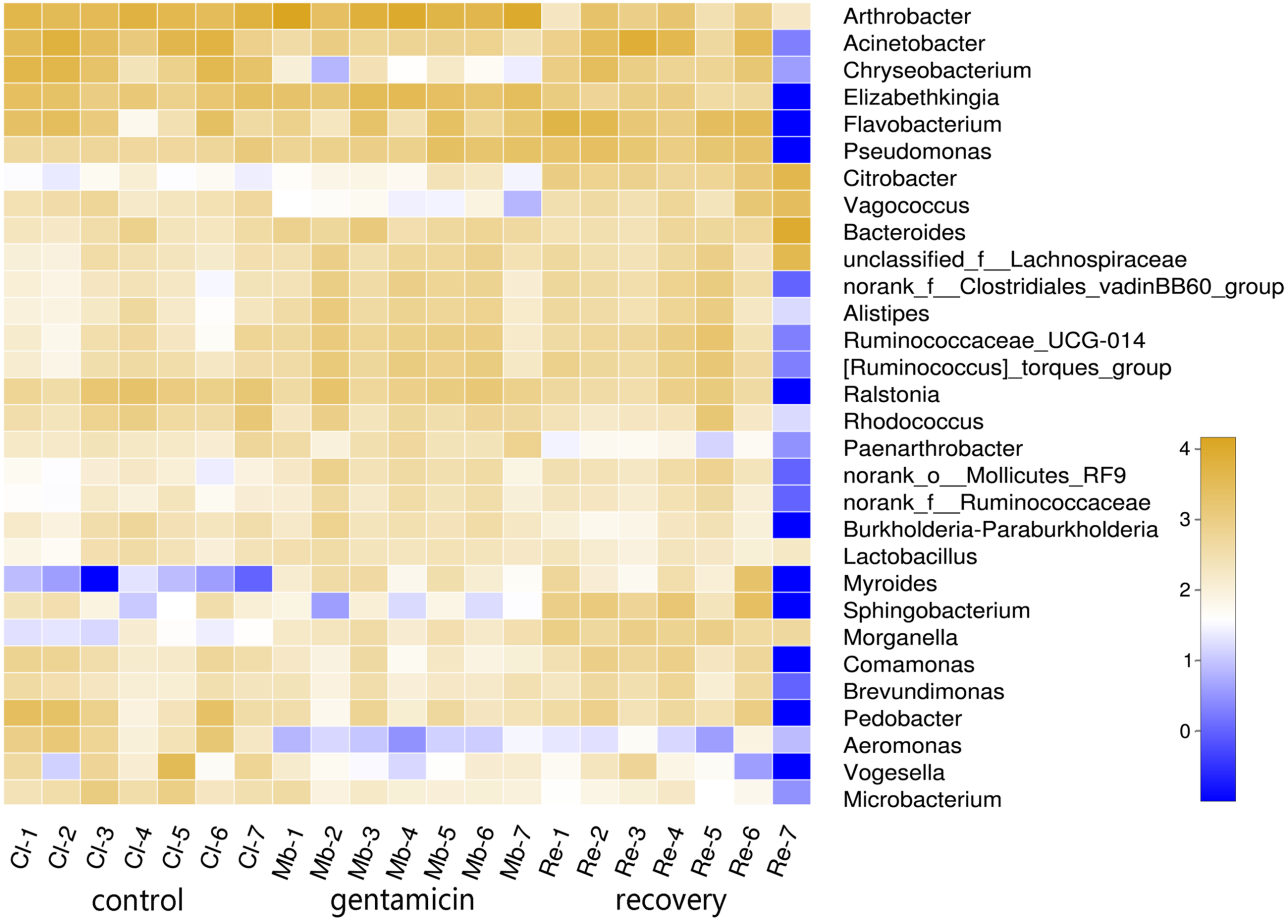
Heatmap of species abundances at the genus level. The lower side and the right side of the heat map show the sample name and genus name, respectively. The intensity of color in the heat map represents the abundance of the genus. Samples in the control group: Cl (1∼7); gentamicin group: Mb (1∼7); and recovery group: Re (1∼7).

To determine the differences in bacterial communities among the three groups, a heatmap was obtained based on the relative abundances of species (for the top 30 species, Fig. 2). As shown in the heatmap, the relative abundances of most bacteria were different, despite some similarities between samples in these three groups.

### Bacterial community structure and distribution at the phylum and genus levels

**Figure 3:**
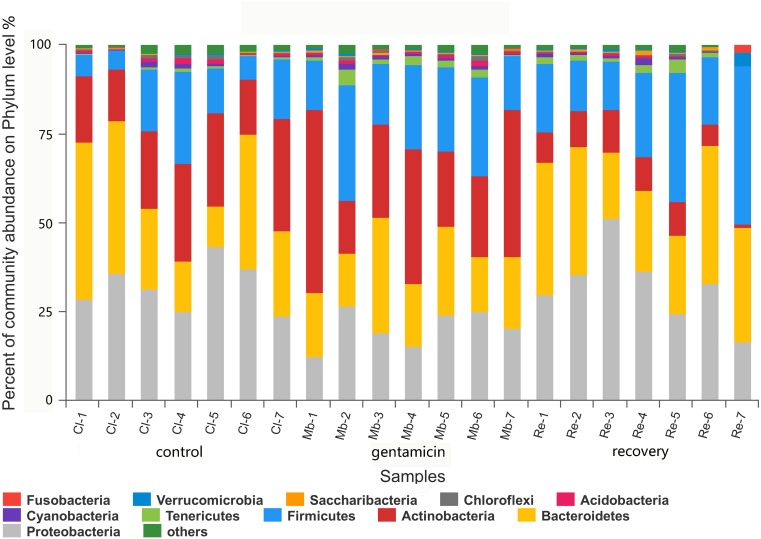
Bacterial community and distribution at the phylum level. The *x*-axis represents the sample name, the *y*-axis represents the proportion of species in the sample, the columns in different colors represent different species, and the length of the column represents the frequency of the species. Samples in the control group: Cl (1∼7); gentamicin group: Mb (1∼7); and recovery group: Re (1∼7).

There were 11 dominant phyla (relative abundance >1.0%) in the samples collected for this study (Fig. 3). In the control group, there were four major phyla (95.59%; average relative abundance >1.0%): Proteobacteria, Bacteroidetes, Actinobacteria and Firmicutes. Most of the sequences in the gentamicin group (95.82%) belonged to the following five phyla: Actinobacteria, Firmicutes, Bacteroidetes, Proteobacteria and Tenericutes. In the recovery group, there were five major phyla (96.28%): Proteobacteria, Bacteroidetes, Firmicutes, Actinobacteria and Tenericutes (Table S1).

**Figure 4:**
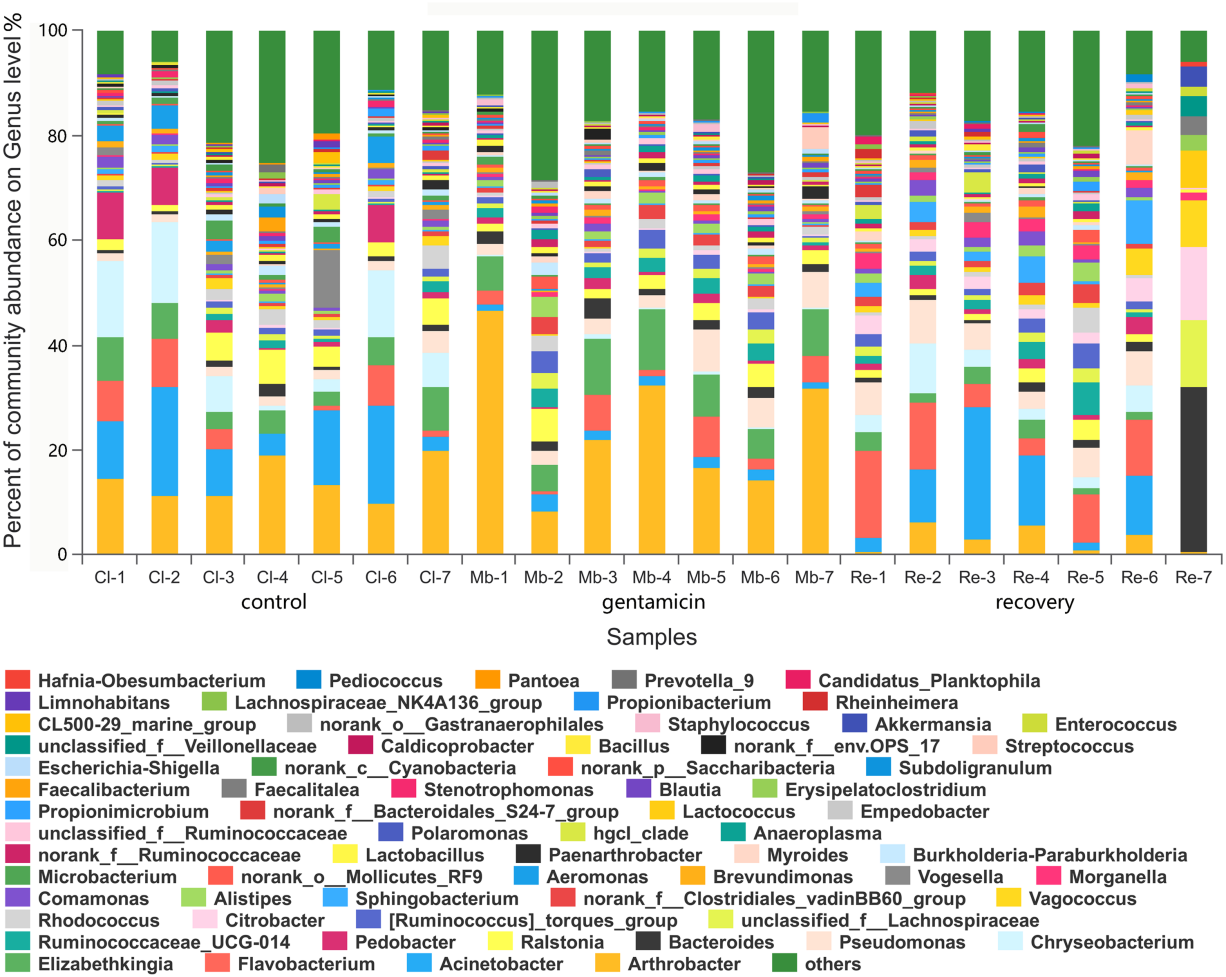
Bacterial community and distribution at the genus level. The *x*-axis represents the sample name, the *y*-axis represents the proportion of the species in the sample, the columns with different colors represent different species, and the length of the column represents the proportion of the species. Samples in the control group: Cl (1∼7); gentamicin group: Mb (1∼7); and recovery group: Re (1∼7).

There were 980 genera in these three groups, including 66 genera with a relative abundance of greater than 1.0% (Fig. 4). The majority of the sequences (average relative abundance >1.0%) in the control group (66.01%) belonged to *Arthrobacter*, *Acinetobacter*, *Chryseobacterium* and *Elizabethkingia*. However, most sequences in the gentamicin group (64.40%) belonged to *Arthrobacter*, *Elizabethkingia*, *Pseudomonas* and *Flavobacterium*. The majority of sequences in the recovery group (70.44%) belonged to *Acinetobacter*, *Flavobacterium*, *Bacteroides* and *Pseudomonas* (Table S2).

**Figure 5:**
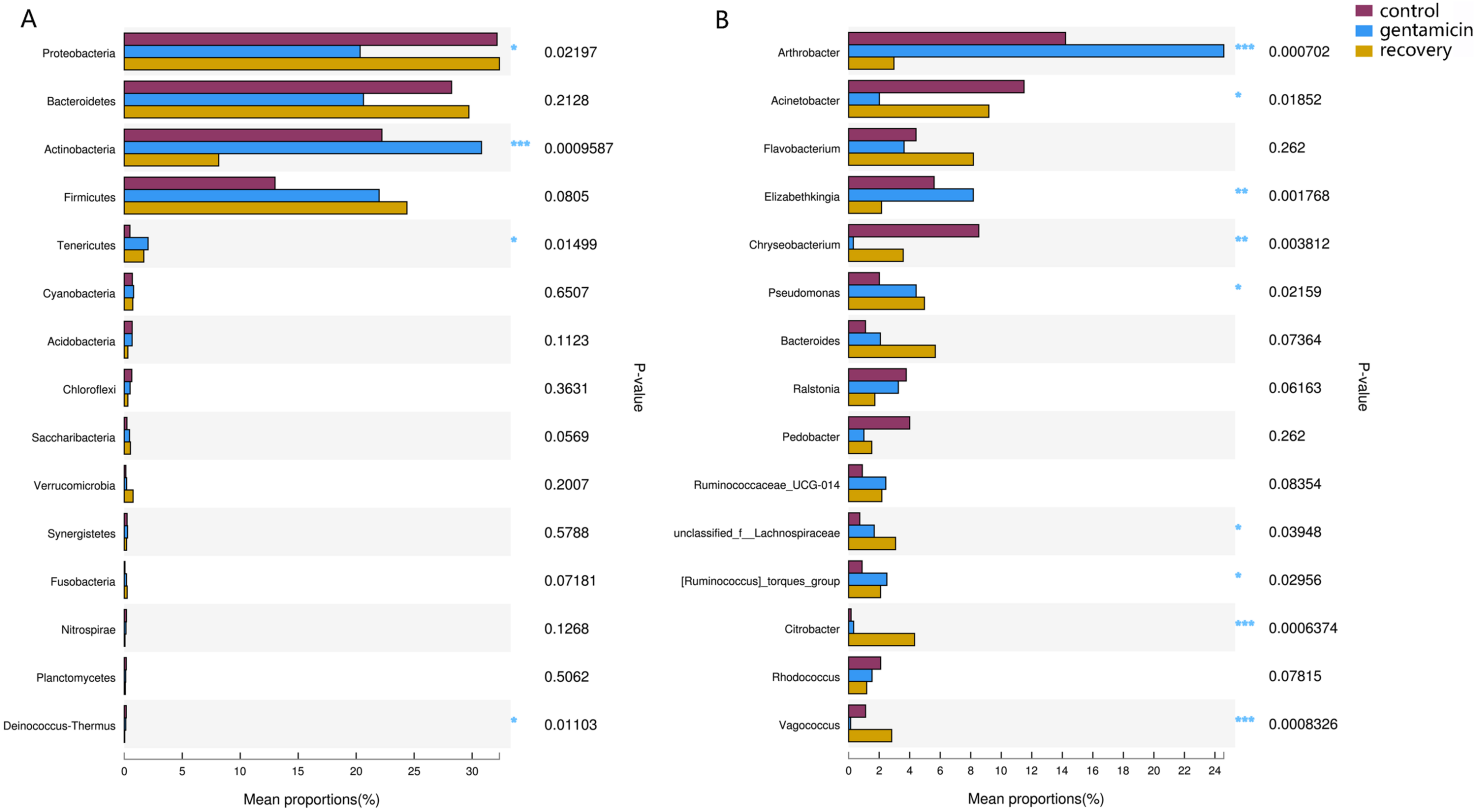
Kruskal–Wallis H test bar plots for analyses at the phylum (A) and genus (B) levels. The *x*-axis indicates species abundance for the sample (expressed as a percentage), the *y*-axis indicates the species name at different classification levels, and different colors indicate different groups. *P*-values are represented, with significant *p*-values denoted by *, 0.01 < *p* ≤ 0.05 *, 0.001 < *p* ≤ 0.01 **, *p* ≤ 0.001 ***.

**Figure 6:**
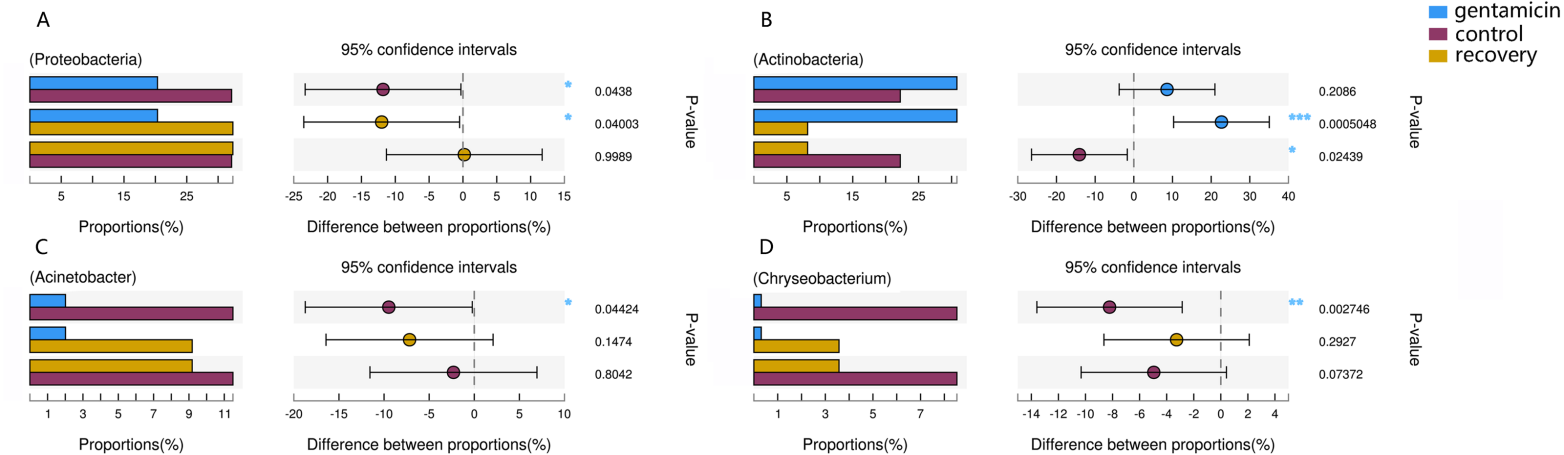
Kruskal–Wallis H test bar plot for the comparison of species between groups. A, Proteobacteria; B, Actinobacteria; C, *Acinetobacter*; and D, *Chryseobacterium*. For each part, the *x*-axis indicates species abundances in each group of samples (expressed as a percentage), the *y*-axis indicates the category of the two pairs, and different colors indicate different groups. The difference between proportions (%) is shown in the figure within the set confidence interval. *P*-values are represented, with significant *p*-values denoted by *, 0.01 < *p* ≤ 0.05 *, 0.001 < *p* ≤ 0.01 **, *p* ≤ 0.001 ***.

Among the top 15 most abundant bacteria, there were significant differences in the relative abundance of four phyla among the three groups (Kruskal–Wallis H test, FDR correction, CI: Scheffer, *p* <0.05; Fig. 5A), one of which was highly significant (*p* <0.01). At the genus level, there were significant differences in the relative abundances of nine genera among the three groups (*p* <0.05; Fig. 5B). The H/Chi-square statistics are reported for the Kruskal-Wallis test in Table 2. The comparison of abundances of key species between groups is shown in Fig. 6 (Kruskal–Wallis H test, FDR correction, CI: Scheffer).

### Potentially pathogenic genera

The distribution of potentially pathogenic genera on the skin of *R. dybowskii* after the gentamicin bath is shown in Table 3. The average number of species belonging to *Aeromonas* and *Chryseobacterium* after the gentamicin bath decreased significantly (Student’s *t*-test; *p* < 0.01, Table 3). After the recovery period, *Chryseobacterium* increased significantly (Student’s *t*-test; *p* < 0.05, Table 3), while the increase in *Aeromonas* was not significant (Student’s *t*-test; *p* > 0.05, Table 3).

**Table 2:** H/Chi-square statistics for the Kruskal–Wallis test.

Phylum	H/Chi-square statistic	Genus	H/Chi-square statistic
Proteobacteria	10.293	*Arthrobacter*	11.963
Bacteroidetes	3.095	*Acinetobacter*	7.978
Actinobacteria	13.900	*Flavobacterium*	2.679
Firmicutes	5.039	*Elizabethkingia*	12.675
Tenericutes	8.883	*Chryseobacterium*	11.319
Cyanobacteria	0.860	*Pseudomonas*	7.671
Acidobacteria	4.095	*Bacteroides*	5.427
Chloroflexi	1.962	*Ralstonia*	1.833
Saccharibacteria	5.753	*Pedobacter*	2.679
Verrucomicrobia	3.464	*Ruminococcaceae_UCG-014*	5.159
Synergistetes	1.160	*unclassified_f__Lachnospiraceae*	6.464
Fusobacteria	5.836	*[Ruminococcus]_torques_group*	7.043
Nitrospirae	4.005	*Citrobacter*	14.726
Planctomycetes	1.083	*Rhodococcus*	5.293
Deinococcus-Thermus	8.964	*Vagococcus*	14.191

**Table 3:** Distribution of potentially pathogenic genera after the antibiotics bath (Student’s *t*-test). Within a row, different small superscript letters indicate significant differences (*p* < 0.05), different capital superscript letters indicate extremely significant differences (*p* < 0.01), and the same superscript letter indicates no significant difference (*p* > 0.05).

Genus	OTU	Control group	Antibiotic group	Recovery group
*Aeromonas*	OTU117, OTU2220	2.293 ± 1.872^A^	0.041 ± 0.028^B^	0.091 ± 0.090^B^
*Citrobacter*	OTU2410, OTU3139	0.159 ± 0.105^a^	0.329 ± 0.270^a^	4.319 ± 4.290^b^
*Chryseobacterium*	OTU75, OTU2705, OTU1673, OTU2706	8.528 ± 5.805^A^	0.307 ± 0.307^Ba^	3.575 ± 2.965^ABb^
*Pseudomonas*	OTU2333, OTU830, OTU2569, OTU55, OTU2484, OTU909, OTU1451, OTU120, OTU2481, OTU2549, OTU849	2.011 ± 0.888^a^	4.422 ± 2.435^b^	4.965 ± 2.662^b^
*Staphylococcus*	OTU13, OTU2975	0.092 ± 0.061^a^	0.418 ± 0.605^a^	0.247 ± 0.352^a^
*Streptococcus*	OTU1627, OTU891, OTU838, OTU2274, OTU946, OTU406	0.265 ± 0.376^a^	0.755 ± 1.531^a^	0.023 ± 0.019^a^

## Discussion

In this paper, high-throughput sequencing was used to detect cutaneous bacterial communities on *R. dybowskii* after treatment with a gentamicin bath and after a recovery period, using a distilled water bath as a control. Differences in the cutaneous bacterial communities among the three groups were evaluated to understand the effects of the gentamicin bath on farmed *R. dybowskii* as well as trends in cutaneous bacterial communities after discontinuation of the bath.

If *R. dybowskii* is infected during the breeding process, it is difficult for drugs to be absorbed through the esophagus because the individual will stop eating soon after onset. Additionally, owing to the large populations of *R. dybowskii*, many methods, such as intramuscular injection and intravenous injection, are difficult to achieve. Therefore, it is particularly important to develop preventive measures for diseases of *R. dybowskii* and to find a drug delivery method suitable for *R. dybowskii*. The skin of *R. dybowskii* has a better ability to absorb drugs than the skin of mammals. Accordingly, an antibiotic bath can be used for effective disease treatment and prevention. The antibiotic used in these experiments was gentamicin, which is widely used in veterinary medicine (Hadfield & Whitaker, 2005). Experiments have shown that gentamicin can be detected in the serum after passing through the skin of a frog by a medicated bath (Riviere, Shapiro & Coppoc, 2010).

The number of OTUs in this study is greater than that in previous studies (Becker et al., 2014; Bataille et al., 2016; Sabino-Pinto et al., 2016). There are two possible reasons for this. The first is because skin peeling was used as the sampling method in this study, whereas swab wiping was used in the other studies. The swab wiping method will definitely miss part of the bacterial community, affecting both the species and content. The second reason is that these frogs were collected from farms, which is a more complicated environment relative to that in which wild *R. dybowskii* live. As such, the frogs are affected by many human factors, such as environmental pollution and bacteria in food, which will increase the number of OTUs (Zhou et al., 2016). In the raw data, the low abundance OTUs are almost all found in the control group. The data in this study has been de-cluttered and optimized, which could represent the true OTU distribution. Moreover, these low abundance OTUs can increase acuity for rare OTU discrimination (Bokulich et al., 2013).

Based on analysis of the bacterial community diversity, the overall difference in alpha diversity caused by the gentamicin bath was not significant, whereas there was a significant difference in beta diversity. The gentamicin bath had inhibitory effects on several potentially pathogenic genera. The change in cutaneous bacterial community diversity was reflected in the differences in composition and relative abundance at the phylum and genus levels.

The PCoA diagram showed that the contributions of PC1 and PC2 were 24.25% and 19.46%, respectively. The intragroup samples in the control group and the gentamicin group were more similar than those in the recovery group. Based on the distribution of sample points in Fig. 1, there were differences in the structure of the bacterial community among these three groups. ANOSIM also indicated significant differences, demonstrating that the antibiotic bath had a significant impact on the beta diversity of the cutaneous bacterial community of *R. dybowskii*. Three groups of individuals were taken from the same farm and fed the same diets; therefore, the gentamicin bath was considered to be the main reason for the change in beta diversity. The number of shared OTUs for the three groups was 1,313, accounting for more than 50% of the OTUs in each of the three groups. The number of species shared by the gentamicin group and the control group was the largest, indicating that the species composition of these two groups was similar, and this result was further supported by the heatmap (Fig. 2). Based on the heatmap, differences in the relative abundance of bacterial species among groups can be seen. The results showed that the effect of the gentamicin bath on skin bacteria changed after the 7–day recovery period, but it was still not restored to the original level (control group).

The dominant phyla in the control group were Proteobacteria, Bacteroidetes, Actinobacteria and Firmicutes. Compared with the control group, the relative abundance of Proteobacteria was significantly lower (Fig. 6A, *p* < 0.01) after the gentamicin bath. After the recovery period, the abundances of Proteobacteria and Bacteroidetes returned to the level observed in the control group. The experimental results showed that the gentamicin bath had an inhibitory effect on Proteobacteria, and the effect disappeared after a recovery period of 7 days.

In the analysis of the dominant genera, the abundances of *Acinetobacter* and *Chryseobacterium* in the control group were ranked second and third, respectively. The relative abundances of the two genera after the gentamicin bath decreased significantly (Figs. 6C and 6D), indicating that the gentamicin bath had an inhibitory effect. Another possible reason for this is niche exclusion. Gentamycin baths can change the micro-environment of bacteria. Other genera have adapted to this change better, thereby strengthening their competitiveness (Mayfield & Levine, 2010) and indirectly inhibiting the growth of these two genera. *Acinetobacter* is widely distributed in the external environment, mainly in water and soil, but is also found in the skin and intestines of healthy people and amphibians; it survives easily in warm and humid environments with strong adhesion (Chen & Lin, 2011; Smani, Mcconnell & Pachón, 2012). At the same time, many opportunist pathogens, such as *Acinetobacter baumannii*, have been isolated from this genus. *Acinetobacter baumannii* can cause serious infections, which can be inhibited by gentamicin, and frog peptides are also active against *Acinetobacter baumannii* (Conlon et al., 2012; Zarrilli et al., 2004). *Chryseobacterium* species are found primarily in soil and water (Kirby et al., 2004). *Chryseobacterium indolgenes* is an etiological agent of red-leg syndrome (Weng, Yang & Wang, 2016). The relative abundances of *Flavobacterium* and *Pedobacter* in the control group were ranked fifth and sixth, respectively. These results showed that the relative abundances of *Flavobacterium* and *Pedobacter* did not change significantly, indicating that the gentamicin bath had no significant inhibitory effect on these taxa. *Flavobacterium* is widely found in soil, water and plants. Several *Flavobacterium* can cause diseases in fish, such as rainbow trout fry syndrome and bacterial cold-water disease caused by *F. psychrophilum* (Bernardet et al., 1996; Hesami et al., 2010). *Pedobacter* has been isolated from soil samples and is also present on the surfaces of amphibians (Margesin & Zhang, 2013; Trinh & Yi, 2016). Studies have shown that *Pedobacter* isolated from *Hemidactylium scutatum* has an inhibitory effect on *Batrachochytrium dendrobatidis in vitro* (Harris et al., 2006). The relative abundance of *Arthrobacter* in the control group was the highest, and it was also the highest in the gentamicin group. These results showed that the genus was not inhibited by the gentamicin bath. Bacteria of the genus *Arthrobacter* are common inhabitants of the soil environment and can be recovered from leaf surfaces. They are known for their ability to degrade a wide variety of organic pollutants (Scheublin & Leveau, 2013). Furthermore, *Arthrobacter* that was isolated from *H. scutatum* has an inhibitory effect on *Batrachochytrium dendrobatidis in vitro* (Harris et al., 2006). The relative abundance of *Elizabethkingia* was higher in the control group than in the other groups and the gentamicin bath did not have an inhibitory effect. *Elizabethkingia* is pathogenic and has four known species, including *Elizabethkingia miricola*, which caused a multi-regional outbreak of meningitis-like cases in a Chinese black spotted frog farm in 2016 (Hu et al., 2017). *Elizabethkingia* was not sensitive to gentamicin in this study. Many of the bacterial colonies on the amphibian skin could act as a barrier and inhibit colonization by other organisms, including fungal pathogens (Mckenzie et al., 2012).

The results of this study showed that the gentamicin bath had a greater effect on the relative abundances of species than on the composition. Potentially pathogenic genera in the samples included *Aeromonas*, *Chryseobacterium*, *Citrobacter*, *Pseudomonas*, *Staphylococcus* and *Streptococcus*. Two potentially pathogenic genera, namely *Aeromonas* and *Chryseobacterium*, were significantly inhibited by the gentamicin bath. *Aeromonas* is an important genus causing damage to amphibians in northeastern China (Xu et al., 2017). In this experiment, the gentamicin bath had a significant inhibitory effect on *Aeromonas*. This research provides a theoretical basis for the use of gentamicin in the breeding of *R. dybowskii*.

## Conclusions

The gentamicin bath had a significant effect on the beta diversity of cutaneous bacterial communities of farmed *R. dybowskii*. After the bath, the dominant phyla were Actinobacteria, Firmicutes, Bacteroidetes, Proteobacteria and Tenericutes; the dominant genera were *Arthrobacter*, *Elizabethkingia*, *Pseudomonas*, *Flavobacterium*, *Ralstonia*, *Bacteroides* and *Acinetobacter*. The gentamicin bath had significant inhibitory effects on the potentially pathogenic genera *Aeromonas* and *Chryseobacterium*.

##  Supplemental Information

10.7717/peerj.8430/supp-1 Figure S1 Sobs (A) and Shannon (B) rarefaction curves for all samples

10.7717/peerj.8430/supp-2 Table S1 Bacterial community and distribution at the phylum level

10.7717/peerj.8430/supp-3 Table S2 Bacterial community and distribution at the genus level

10.7717/peerj.8430/supp-4 Supplemental Information 4 Sequence data

## Additional Information and Declarations

### Field Study Permissions

The following information was supplied relating to field study approvals (i.e., approving body and any reference numbers): Fenglin Gao is the owner of the *Rana dybowskii* farm and gave authorization to access his land.

### Data Availability

The following information was supplied regarding data availability: Data is available at NCBI BioProject: PRJNA509105, PRJNA509098.
