# Supplementary figures and images for: Comparative analysis of cutaneous bacterial communities of farmed Rana dybowskii after gentamycin bath
Source: PeerJ. 2020 Jan 20;8:e8430. doi: 10.7717/peerj.8430 (PMC6977512; doi:10.7717/peerj.8430)

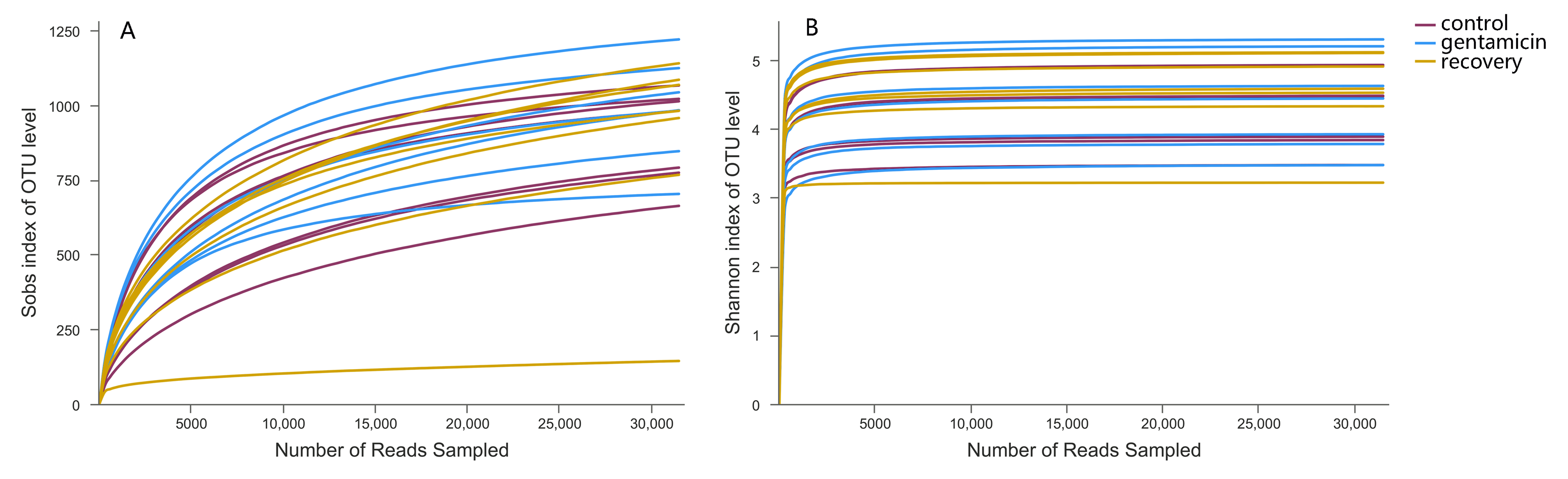

Supplement: Figure S1 [file peerj-08-8430-s001.png]
