# Supplementary material for: Comparative analysis of cutaneous bacterial communities of farmed Rana dybowskii after gentamycin bath
Source: PeerJ. 2020 Jan 20;8:e8430. doi: 10.7717/peerj.8430 (PMC6977512; doi:10.7717/peerj.8430)
Supplement: Table S1 [file peerj-08-8430-s002.docx]

| Groups | Average relative abundance |
| --- | --- |
| control | Proteobacteria (32.15%), Bacteroidetes (28.23%), Actinobacteria (22.22%), and Firmicutes (13.00%) |
| gentamicin | Actinobacteria (30.81%), Firmicutes (21.98%), Bacteroidetes (20.63%), Proteobacteria (20.34%), and Tenericutes (2.05%) |
| recovery | Proteobacteria (32.35%), Bacteroidetes (29.72%), Firmicutes (24.38%), Actinobacteria (8.14%), and Tenericutes (1.69%) |
