# Supplementary material for: Comparative analysis of cutaneous bacterial communities of farmed Rana dybowskii after gentamycin bath
Source: PeerJ. 2020 Jan 20;8:e8430. doi: 10.7717/peerj.8430 (PMC6977512; doi:10.7717/peerj.8430)
Supplement: Table S2 [file peerj-08-8430-s003.docx]

| Groups | Average relative abundance |
| --- | --- |
| control | *Arthrobacter* (14.21%), *Acinetobacter* (11.49%), *Chryseobacterium* (8.53%), *Elizabethkingia* (5.60%), *Flavobacterium* (4.41%), *Pedobacter* (4.00%), *Ralstonia* (3.77%), *Aeromonas* (2.29%), *Vogesella* (2.43%), *Rhodococcus* (2.09%), *Pseudomonas* (2.01%), *Microbacterium* (1.61%), *Comamonas* (1.34%), *Vagococcus* (1.11%), and *Bacteroides* (1.10%) |
| gentamicin | *Arthrobacter* (24.58%), *Elizabethkingia* (8.17%), *Pseudomonas* (4.42%), *Flavobacterium* (3.63%), *Ralstonia* (3.25%), [Ruminococcus]_torques_group (2.50%), *Ruminococcaceae_UCG-014* (2.43%), *Bacteroides* (2.08%), *Acinetobacter* (2.01%), *Alistipes* (1.93%), norank_f __Clostridiales_vadinBB60_group (1.84%), unclassified_f __Lachnospiraceae (1.67%), *Rhodococcus* (1.53%), *Paenarthrobacter* (1.18%), norank_o__Mollicutes_RF9 (1.14%), and *Burkholderia-Paraburkholderia* (1.02%) |
| recovery | *Acinetobacter* (9.18%), *Flavobacterium* (8.17%), *Bacteroides* (5.68%), *Pseudomonas* (4.97%), *Citrobacter* (4.32%), *Chryseobacterium* (3.57%), *Sphingobacterium* (3.25%), unclassified_f__Lachnospiraceae (3.07%), *Arthrobacter* (2.97%), *Vagococcus* (2.82%), *Morganella* (2.23%), *Ruminococcaceae_UCG-014* (2.17%), *Elizabethkingia* (2.15%), [Ruminococcus]_torques_group (2.09%), *Ralstonia* (1.71%), norank_f__Clostridiales_vadinBB60_group (1.61%), *Myroides* (1.48%), *Lactococcus* (1.20%), and *hgcI_clade* (1.12%) |
