# Supplementary material for: Comparative analysis of cutaneous bacterial communities of farmed Rana dybowskii after gentamycin bath
Source: PeerJ. 2020 Jan 20;8:e8430. doi: 10.7717/peerj.8430 (PMC6977512; doi:10.7717/peerj.8430)
Supplement: Supplemental Information 4 [file peerj-08-8430-s004.docx]

Sequence data have been deposited on the BioProject database：

Water group：

https://www.ncbi.nlm.nih.gov/bioproject/?term=PRJNA509105

SubmissionID: SUB4899397

BioProject ID: PRJNA509105

Gentamycin group and recovery group：https://www.ncbi.nlm.nih.gov/bioproject/?term=PRJNA509098

SubmissionID: SUB4898399

BioProject ID: PRJNA509098

All other data are available upon request from the authors.
